# A Remote Maintenance Support Method for Complex Equipment Based on Layered-MVC-B/S Integrated AR Framework

**DOI:** 10.3390/s25195935

**Published:** 2025-09-23

**Authors:** Xuhang Wang, Qinhua Lu, Jiayu Chen, Dong Zhou

**Affiliations:** 1 College of Civil Aviation, Nanjing University of Aeronautics and Astronautics, Nanjing 210016, China; wangxuhang@nuaa.edu.cn (X.W.); lu_qinhua@nuaa.edu.cn (Q.L.); 2 School of Reliability and Systems Engineering, Beihang University, Beijing 100191, China; zhoudong@buaa.edu.cn

**Keywords:** augmented reality, remote maintenance support, collaborative maintenance, MVC architecture, B/S architecture

## Abstract

Augmented reality (AR)-based assisted maintenance methods are effective in completing simple equipment maintenance tasks. However, complex equipment typically requires multi-location remote collaboration due to structural complexity, multiple fault states, and high maintenance costs, significantly increasing maintenance difficulty. This paper therefore proposes a remote maintenance support method for complex equipment based on layered-MVC-B/S integrated AR framework (IAR-RMS). First, clearly define the maintenance content and workflow for multi-person remote collaboration and conduct an in-depth analysis of process control within the task workflow to avoid incomplete or unsystematic maintenance guidance information and processes. Second, analyze collaborative management from the perspectives of maintenance role conflicts and maintenance operation conflicts and implement on-demand permission control and operation sequence management to ensure the timeliness and user-friendliness of multi-person collaboration. Then, integrate the layered architecture, MVC, and B/S architecture to construct a remote maintenance support (RMS) model based on an integrated architecture system, ensuring the reliability and timeliness of the model. Finally, demonstrate the main functional modules of the RMS task process, and use power system disassembly and assembly as an experiment to validate the effectiveness and generalizability of the proposed IAR-RMS method. The results indicate that the proposed IAR-RMS method can effectively realize maintenance support tasks in multi-person remote collaboration scenarios.

## 1. Introduction

With the rapid development of mechanization, electrification, and digitization in manufacturing, a well-established technical system has been developed for the production and manufacturing of complex equipment [1,2,3,4]. However, the corresponding maintenance and support capabilities still lag behind, falling short of the increasingly higher demands for efficiency and precision in maintaining such equipment [5,6,7,8]. In sectors like aerospace, traditional maintenance approaches face significant limitations due to factors including strong engineering applicability constraints and restricted access to maintenance sites [9,10,11]. Crucially, during the engineering design phase, maintenance design often cannot rely on physical prototypes, severely limiting quality control over maintenance process implementation [12,13]. Therefore, complex equipment maintenance urgently necessitates a remote, real-time, and collaborative technological approach to enhance existing maintenance practices.

With the continuous advancement of augmented reality (AR) technology, significant progress has been made in equipment maintenance analysis and methodologies, particularly for complex multi-person collaborative maintenance scenarios [14,15,16,17]. AR integrates virtual information with the real-world environment through techniques such as 3D modeling and human–computer interaction to superimpose computer-generated models, actions, and interaction information onto physical settings [18,19,20,21]. Yang et al. developed a more realistic virtual tactile disassembly platform, enhancing disassembly training and learning efficiency [22]. Liu et al. introduced a human–machine collaborative maintenance method based on multi-sensory perception, enabling maintenance technicians to interact with collaborative robots for auxiliary tasks without spatial or human factor constraints [23]. However, research on collaborative maintenance technologies for complex equipment remains insufficient. Particularly in practical maintenance support tasks, existing approaches have not yet effectively enhanced maintenance efficiency or reduced time costs. Therefore, there is an urgent need to develop augmented reality-based remote maintenance support technologies.

To enhance the real-time performance and stability of AR technology in maintenance guidance processes, AR technology has been integrated with collaborative software architectures to enable a new generation of AR-based collaborative maintenance systems [24]. Benbelkacem et al. proposed an MVC-3D design pattern for developing AR interfaces, which reduces complexity in model components [25]. Chen et al. introduced a hybrid architecture based on C/S and B/S structures, thereby improving software robustness and system management efficiency [26]. However, AR-based collaborative maintenance tasks impose high demands on system architectures, which are often difficult to fully meet with a single architecture. The typical MVC architecture, for instance, has a complex structure and is prone to controller redundancy. Meanwhile, the B/S architecture heavily depends on network and server resources, creating security challenges. Therefore, to satisfy the stringent performance requirements of assisted maintenance tasks, it is necessary to develop an integrated system architecture that combines the advantages of multiple architectural paradigms.

Although AR-based maintenance assistance is a current research hotspot, its application in complex equipment maintenance faces challenges, particularly in remote multi-collaborator scenarios [27]. (1) Incomplete guidance information and processes. Complex equipment maintenance typically requires multi-party collaboration (equipment users and manufacturers), demanding significant resources. Current solutions lack timeliness and fail to meet actual maintenance needs. (2) Maintenance collaboration conflicts. During multi-person collaboration, simultaneous operations cause conflicts, leading to operational errors that compromise equipment safety and reliability. (3) Deficient traditional collaboration models. Traditional approaches are limited by single-point knowledge constraints, lack real-time multi-expert platforms, and suffer from fragmented information and chaotic status management, resulting in low efficiency in complex scenarios. Therefore, AR-assisted maintenance urgently requires multi-level, multi-angle, multi-dimensional RMS technology to address increasingly complex maintenance systems.

To address the challenges in multi-person remote collaborative maintenance, this paper proposes a remote maintenance support method for complex equipment based on layered-MVC-B/S integrated AR framework (IAR-RMS). The proposed method establishes a comprehensive remote maintenance collaboration framework, enabling accelerated task completion for distributed personnel and enhancing complex equipment maintenance efficiency. The main contributions of this paper are as follows:

(1) A remote maintenance support (RMS) process design and analysis method is introduced. This approach organizes and categorizes the required maintenance guidance information to facilitate RMS process analysis. Building on this foundation, an overarching RMS task framework is constructed to clarify the flow of maintenance information during operations. Finally, RMS task process control is implemented to regulate interactions throughout the support cycle.
(2) A collaborative management approach for RMS is proposed to mitigate operational conflicts during multi-person collaboration. This method classifies collaborative conflicts into role conflicts and task conflicts. Maintenance role conflicts are distinguished on the basis of the divergent tasks and functions of the participants concerned, with the relevant permissions specified. Maintenance task conflicts address the issue of concurrent operations on the same object, where two sequence management strategies are implemented to prevent conflicts.
(3) An RMS model based on layered-MVC- B/S integrated architecture is proposed to support multi-person collaborative RMS tasks. The model defines the interrelationships between the three integrated architectures and explains their mechanisms to ensure the reliability and timeliness of the RMS model.

The organization of this paper is divided below. Section 2 presents a comprehensive overview for the proposed IAR-RMS method. Section 3 discusses experimental analysis results to showcase the effectiveness for our proposed method. Section 4 summarizes the conclusions.

## 2. The Proposed IAR-RMS Method for Complex Equipment

To address multi-person collaborative remote maintenance challenges, this paper proposes an IAR-RMS method for complex equipment, enabling the rapid execution of remote multi-person maintenance tasks while ensuring maintenance efficiency. First, to address incomplete maintenance guidance information and processes, an RMS process design and analysis method is proposed. This method clarifies multi-person remote collaborative workflows and establishes the foundation for subsequent implementation. Second, an RMS collaborative management approach is introduced to mitigate role and task conflicts during multi-person collaboration. Finally, a layered-MVC-B/S integrated architecture-based RMS model is implemented to support multi-person collaborative tasks with ensured reliability and timeliness.

### 2.1. RMS Process Design and Analysis

To address incomplete maintenance guidance information and processes, an RMS process design and analysis method is proposed. This method systematically defines multi-person remote collaborative maintenance workflows, providing the foundation for subsequent collaborative control and RMS models. First, RMS task process guidance information—including key parameters such as maintenance content and fault conditions—is organized and classified. Second, an RMS task process framework is constructed to clarify information flow and interaction during maintenance tasks. Finally, process control mechanisms are examined to ensure efficient and stable operations.

#### 2.1.1. RMS Guidance Information Classification

Maintenance guidance information serves as the foundation for information interaction during the RMS process. It encompasses the physical parameters, structural composition, maintenance content, maintenance resources, and fault condition required by the relevant personnel involved in the maintenance process. This information critically influences whether the RMS process can be initiated, as illustrated in Table 1.

The RMS process is accomplished through the flow and transmission of various types of maintenance guidance information and its interaction with personnel. By integrating AR technology, the MVC, and the B/S architecture, the display and presentation of maintenance guidance information are managed. The RMS methodology for complex equipment utilizes AR spatial scene visualization technology and human–computer interaction to enable involved personnel to receive, read, transmit, and share maintenance guidance information. Simultaneously, it employs networked methods to provide visualized presentation of personnel and information from different ends. This enhances technical coordination and user-friendliness during RMS usage, achieving enhanced information presentation throughout the maintenance process.

#### 2.1.2. RMS Task Flow Framework

The execution of general, simple RMS tasks first requires the identification of the physical equipment entity and the involved personnel. The personnel involved in RMS tasks typically include on-site maintenance technicians and remote experts. Subsequently, human–computer interaction is performed between the physical equipment entity and its corresponding virtual model to facilitate the effective transmission of information throughout the RMS task process, thereby forming a complete closed-loop task process. However, due to the inherent structural complexity, diverse failure states, and unique working environments of complex equipment, their RMS task workflows become intricate, often containing redundancies, resulting in significantly increased overall difficulty for RMS. Therefore, this section analyzes the RMS task process for complex equipment. Based on the maintenance manuals and relevant technical documentation for complex equipment, the maintenance content is planned, and an RMS task process framework is constructed. This framework aims to enhance the operational efficiency of RMS tasks.

Based on the maintenance manuals and relevant technical documentation for complex equipment, the maintenance content for RMS is systematically planned and designed. This content includes the maintenance methods, levels, task types, and actions for the complex equipment. This process establishes a clear, standardized, and efficient RMS workflow. It guides maintenance technicians in accurately and safely completing maintenance support tasks for complex equipment, as detailed in Table 2.

**Figure 1:**
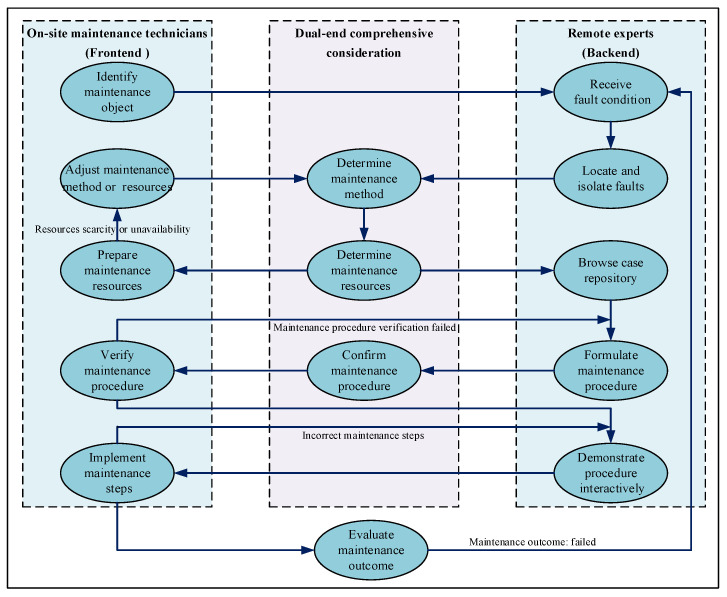
The RMS task process framework.

During the RMS process, the absence of a unified framework results in negligible improvement in operational efficiency. Therefore, based on the defined RMS maintenance content and incorporating practical maintenance procedures, a standardized RMS process framework was constructed, as shown in Figure 1.

First, on-site maintenance technicians transmit the fault information of the complex equipment requiring maintenance to remote experts. Both parties then collaboratively perform fault location and isolation. Second, they jointly determine the maintenance level and maintenance method for the equipment. This RMS is typically performed at the organizational and intermediate levels. Then, based on the fault condition, maintenance level, and maintenance method, the required maintenance resources are identified, and maintenance technicians select them from the resource repository. Finally, leveraging existing cases and maintenance steps, both parties comprehensively deliberate and present the solution to the on-site technicians via various interactive methods, such as visual media. Remote experts simultaneously provide guidance and supervision, enabling the collaborative completion of the complex equipment RMS task.

#### 2.1.3. RMS Process Control Analysis

Maintenance Procedure Control constitutes an essential component of the RMS process. Serving as the structural framework for RMS guidance information, it enables procedure-oriented interaction management for personnel. By integrating workflow management technology and AR technology, maintenance procedures from multiple terminals are consolidated into a unified interface with modification/editing capabilities. This allows personnel across roles to efficiently utilize RMS procedures for managing maintenance steps.

**Figure 2:**
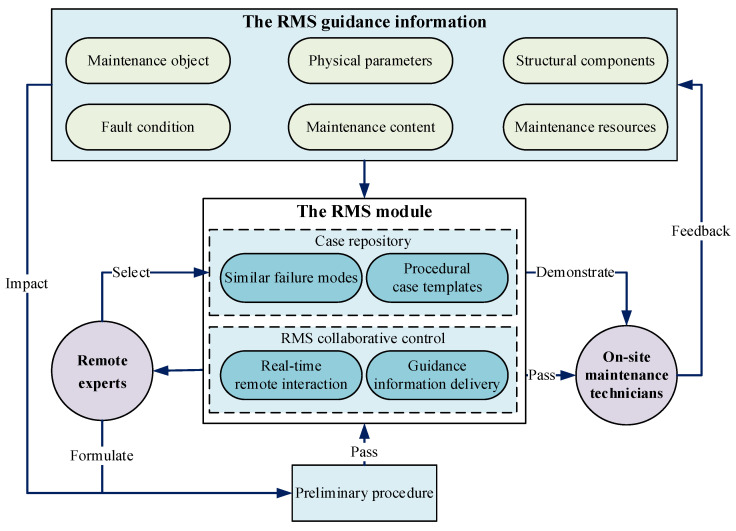
The RMS procedure control framework.

Within RMS procedure control, RMS guidance information functions as the interactive foundation for procedure execution. Maintenance technicians and expert personnel achieve guidance information delivery and interaction through the RMS model, which serves as the core management unit for the entire maintenance task. The detailed RMS procedure control process is illustrated in Figure 2.

First, on-site maintenance technicians transmit RMS guidance information to remote experts through the RMS model. Second, remote experts formulate a Preliminary procedure based on the maintenance guidance information and transmit it to front-line maintenance technicians through the remote collaboration control sub-module. Then, experts use the case repository sub-module to select similar fault cases and demonstrate them to maintenance technicians. Finally, maintenance technicians implement maintenance steps based on the information obtained from the RMS model and provide real-time guidance feedback, thereby synchronously adjusting the maintenance process to achieve the goal of RMS process control.

### 2.2. RMS Collaborative Management Analysis

During RMS collaborative management, conflicts frequently arise when multiple users concurrently perform core maintenance tasks. To ensure smooth multi-user maintenance operations, addressing these conflicts is essential. From the user perspective, conflicts primarily manifest in two dimensions: role allocation and operation execution. These dimensions are analyzed in detail below.

#### 2.2.1. Maintenance Role Conflict Management

Maintenance roles comprise two core components: the maintenance role of architecture within the collaborative system and the distinct permissions assigned to each role.

(1) Analysis of maintenance role system

**Figure 3:**
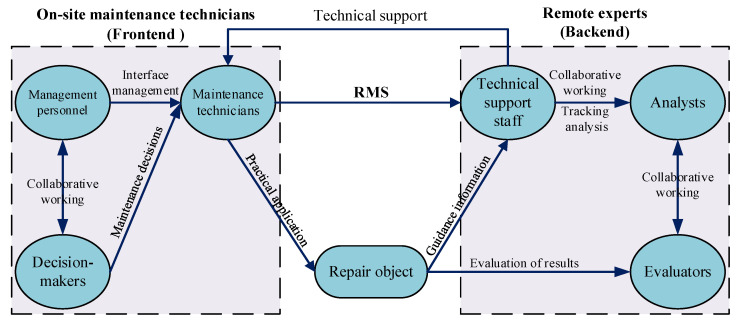
Maintenance of inter-role relationships.

Originating from the core architectural requirements, the role system necessitates analysis to define distinct functional roles: frontline maintenance technicians and remote backend experts. These are further classified as maintenance technicians, technical support staff, management personnel, decision-makers, evaluators, and analysts. The inter-role relationships are illustrated in Figure 3.

Within this framework, maintenance technicians execute on-site maintenance tasks and may require guidance due to limited familiarity with equipment architecture or procedures. Technical support staff provide specialized expertise in equipment configuration, fault diagnosis, and maintenance protocols; while not directly performing maintenance, they diagnose issues using real-time field data and deliver technical guidance to technicians. Management personnel coordinate maintenance resources, optimize technician-support pairings based on fault characteristics, and ensure efficient collaboration between frontline and remote teams. Decision-makers develop comprehensive maintenance strategies, assign tasks using fault-mode analysis and solution databases, and authorize execution plans. Evaluators validate post-maintenance equipment functionality against standardized performance metrics, identify residual deficiencies, provide feedback loops, and determine production readiness. Analysts monitor system-wide maintenance workflows and network efficiency, conducting cost–benefit analyses and multidimensional evaluations of collaborative performance.

According to the RMS collaboration process, preliminary responses to the needs of the above personnel can be made, and a table of actual work requirements for different personnel can be formed, as shown in Table 3. Different personnel roles will lead to different focuses in the network. From the perspective of requirements, port design can make the use of each user more realistic. In the Table 3 and Table 4, the symbol “√” is used to indicate the requirements and permission allocations of each maintenance role, providing a clear presentation.

(2) Maintenance role permission settings management

During design, different permissions are assigned to different users. For example, personnel responsible for specific sections can only operate objects within those sections. In the RMS collaboration system, different user roles have explicit permission requirements based on their responsibilities. Actual maintenance technicians and technical support staff require virtual scene interaction and control permissions, while managers and decision-makers do not need such operational permissions. Evaluators primarily assess the overall smoothness of the collaboration process and maintenance work, rather than recording maintenance details. Analysts focus on issues arising during collaboration, new fault patterns, and subsequent improvement plans. Specific role permissions are detailed in Table 4.

#### 2.2.2. Maintenance Operation Conflict Management

When performing operations on virtual models or editing maintenance processes for AR spatial displays, conflicts may arise when multiple roles attempt to access the same object simultaneously. To address this challenge, this paper adopts a temporal management approach, categorized as object oriented and user oriented. Based on activation sequence, permissions for either the target object or initiating user are locked.

**Figure 4:**
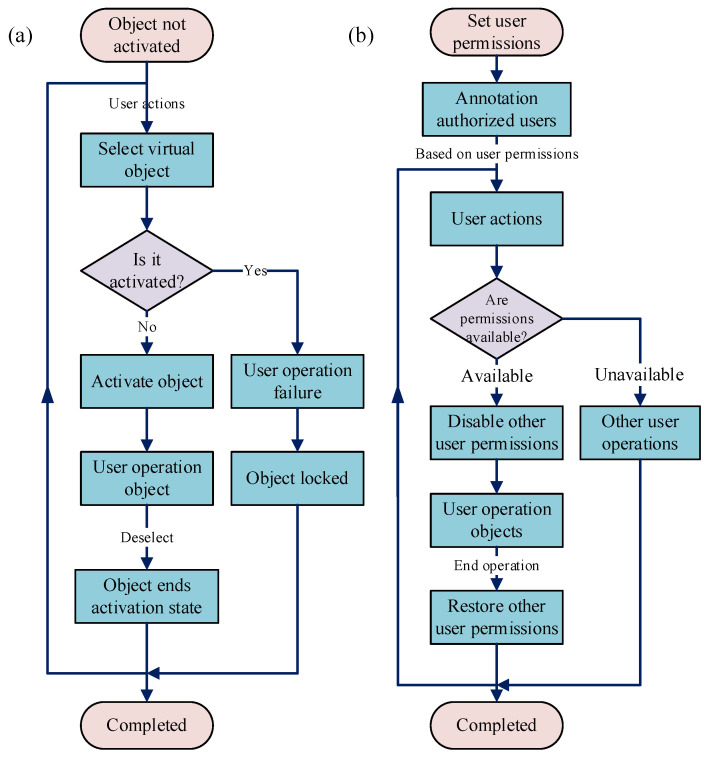
Maintenance operation time series management: (**a**) object-oriented time-series management; (**b**) user-oriented time-series management.

In object-oriented time-series management, all objects and their sub-objects within the virtual space are initially inactive. Any user with virtual–physical interaction permissions can operate on them, as shown in Figure 4a. When a user selects an object, it transitions from an inactive to an active state and appears in all users’ display interfaces. Simultaneously, control of the active object is granted to the user who first triggered activation, based on chronological sequence, ensuring exclusive operation rights.

In user-oriented time-series management, the system first defines user operation permissions and establishes an authorized user database for each object, as shown in Figure 4b. When a database-registered user initiates object operation, the system immediately revokes other users’ corresponding operation permissions for that object until completion, ensuring single-user operational exclusivity at any given time.

Both object-oriented and user-oriented time-series management rely on system time to determine user activation order. The advantages of this approach include the following: the system’s automated decision-making is accurate, effectively preventing conflicts arising from concurrent operations; and the time-locking mechanism is user-intuitive and robust, preventing disruptions to the operator’s interactive demonstration from other users. Comparatively, user-oriented strategies require finer-grained permission classification and definition, alongside storage of user-object correspondence, resulting in higher system complexity. Therefore, object-oriented time-series management sees broader adoption.

### 2.3. RMS Model Based on Layered-MVC-B/S Integrated Architecture

To address the deficiencies of traditional remote collaboration models, an RMS model based on layered-MVC-B/S integrated architecture is proposed for use in multi-person collaborative tasks. First, the layered architecture, MVC, and B/S architectures are integrated, forming a unified architectural system. Second, functional relationships between the MVC architecture and sub-modules within the layered architecture are defined to clarify model interactions. Finally, operational processes of the MVC/B/S architecture are delineated to ensure RMS model reliability and timeliness.

#### 2.3.1. Overall Framework of the RMS Model Based on Integrated Architecture

The RMS model integrates a layered architecture with the MVC and B/S architectures to form a comprehensive system architecture. This integrated architecture achieves systematic decoupling and functional modularization across the entire chain from the user interface to backend services, which effectively overcomes the limitations inherent to single-architecture approaches in complex multi-user collaborative scenarios. Building upon this foundation, the model leverages the cross-platform advantage of the B/S mode while incorporating the client-side MVC pattern to enhance real-time interactive processing capabilities. Simultaneously, the layered design ensures high cohesion and extensibility of backend systems, thereby significantly enhancing the real-time performance and operational fluency of remote AR-assisted collaborative operations.

**Figure 5:**
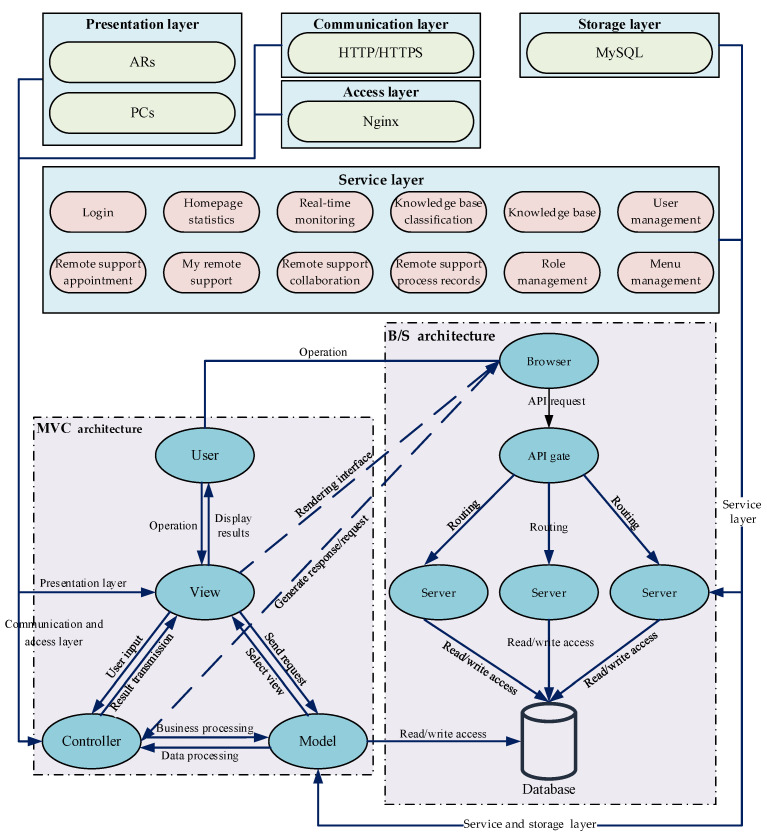
Overall framework of the RMS model based on integrated architecture.

The layered architecture within this framework comprises the presentation, communication, access, storage, and service layers. The presentation layer displays interfaces on AR glasses and PCs, transmitting information to the View layer of the MVC pattern for rendering. The communication layer employs HTTP/HTTPS for protocol conversion and data transmission. The access layer utilizes Nginx for load balancing and interfaces with the Controller layer to prevent single-point failures. The storage layer uses MySQL for structured data persistence and provides data to the Model layer. The service layer deploys business functions separately and combines Service layer components with the Model layer to achieve full functionality. The integrated architecture system is detailed in Figure 5.

#### 2.3.2. MVC Architecture Design and Analysis

**Figure 6:**
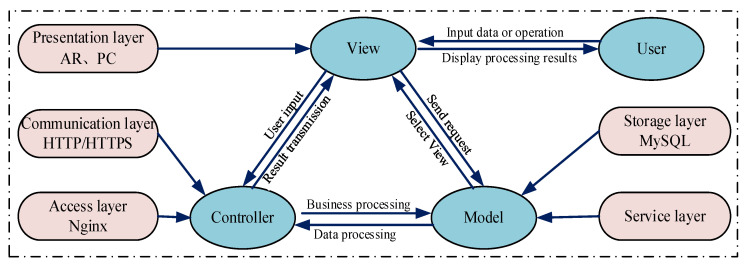
MVC architecture framework of the RMS model.

MVC is a classic software design pattern extensively employed in web development. Its architecture comprises three collaborative yet independent components: the Model, the View, and the Controller. The Model handles business rule encapsulation and data logic processing; the View manages data presentation via the user interface; the Controller mediates user input by invoking Model operations and relaying the results to the View for display. This strict separation of concerns ensures high cohesion and loose coupling among components, which significantly reduces inter-module dependencies, minimizes code redundancy, and improves software quality and maintainability. Within the RMS model, the MVC framework governs three primary functional aspects, and its workflow is illustrated in Figure 6.

(1) The Model layer processes the business logic of maintenance data for the RMS model. It handles maintenance object information, user data, and maintenance guidance data, including storage, verification, and updating operations.
(2) The View layer implements dual-end interface rendering for the RMS display layer. It presents model data through user interfaces, such as displaying 3D models on AR glasses or RMS interfaces on PCs.
(3) The Controller layer processes user input received from the View and updates the Model and View based on these operations. For example, it receives user actions while verifying resources and permissions.

Based on the MVC architecture, the RMS model achieves effective decoupling of data processing, display layer, and interaction control. This separation significantly enhances system modularity, clarifies module structure, and enables streamlined maintenance and functional expansion. Furthermore, MVC implementation strengthens module security, operational stability, and portability, establishing a robust technical foundation for reliable operation and efficient management of RMS services.

#### 2.3.3. B/S Architecture Design and Analysis

The Browser/Server (B/S) architecture extends the traditional two-tier Client/Server (C/S) model into a three-tier structure. This model centralizes core functionality on the server, streamlining system development, maintenance, and usage. Systems based on the B/S architecture utilize browsers as clients, with all core processing occurring on the server. This approach simplifies system upgrades and maintenance. Furthermore, these systems require no specialized client-side installation beyond a web browser, implement most logic on the server side, and primarily use the front end for data presentation. This architecture consequently enhances flexibility for RMS module implementation.

**Figure 7:**
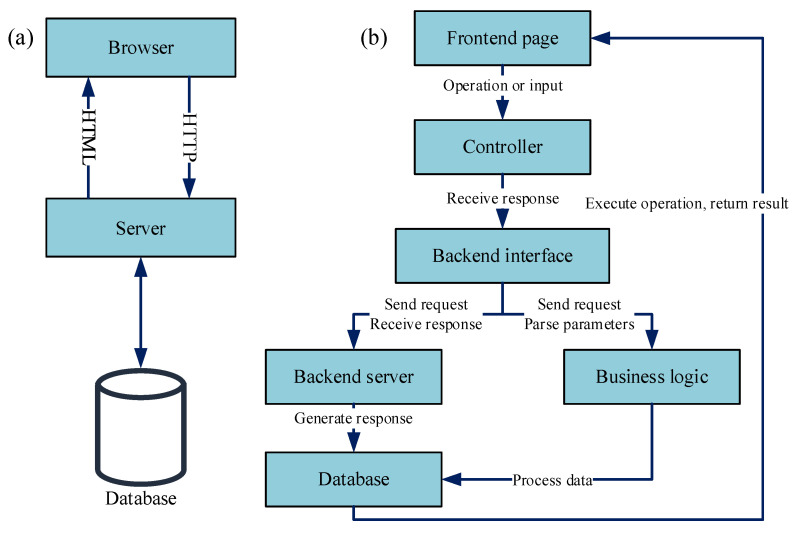
B/S development framework flowchart: (**a**) B/S architecture infrastructure diagram; (**b**) B/S architecture design framework of the RMS model.

A system designed based on the B/S architecture can be divided into three layers: the presentation layer, the logic layer, and the persistence layer. These layers interact with each other. The specific structure of the B/S architecture is shown in Figure 7a.

The presentation layer constitutes the top-level interface, directly receiving user commands via browsers and relaying them to the business logic layer. The logic layer functions as the core processing unit. It is responsible for executing business operations, handling presentation layer requests, returning processed data, and initiating database operations when required. The persistence layer manages data storage and implements CRUD (Create, Read, Update, Delete) operations. This layered division establishes a collaborative workflow encompassing user interaction, business processing, and data persistence.

The RMS model utilizes a B/S architecture with a three-tier structure comprising the browser side, the application service layer, and the data storage layer. The browser side is responsible for displaying the user interface, receiving user actions and inputs, and submitting them to the application service layer. The application service layer processes business logic. It receives commands from the browser side, executes the relevant operations, and passes the processing results or subsequent requests to the data storage layer. The data storage layer interacts with the database to perform operations such as persistent storage and data updates. The detailed process is illustrated in Figure 7b.

## 3. Illustrative Examples

To validate the effectiveness of the proposed IAR-RMS method, this study integrates prior research foundations with maintenance collaboration technology. We establish a collaborative maintenance system prototype and conduct case applications, specifically addressing multi-location, multi-person maintenance challenges to ensure seamless RMS task execution.

### 3.1. RMS System Functiona Showcase

As a core component for complex equipment remote support, the RMS system implements an integrated architecture that combines a layered design with the MVC and B/S frameworks. This enables the interconnection of AR systems and PCs, ensuring real-time interaction between on-site maintenance technicians and remote experts. The module incorporates 12 functional sub-modules: login, homepage statistics, my remote support, remote support appointment, remote support process records, real-time monitoring, remote support collaboration, knowledge base classification, knowledge base, user management, role management, and menu management. The key sub-modules include the following:

(1) Login sub-module

**Figure 8:**
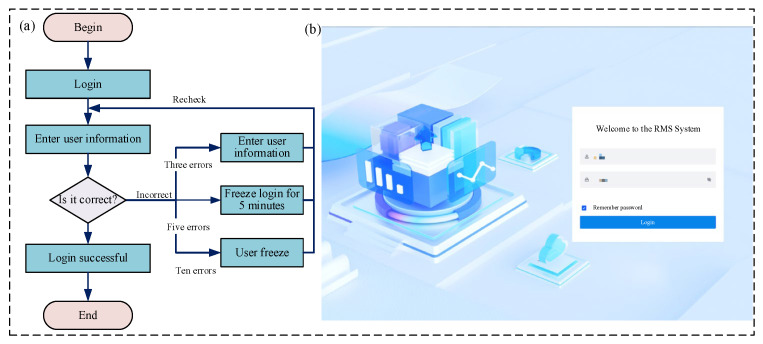
Login sub-module: (**a**) login sub-module functional flowchart; (**b**) login sub-module display diagram.

The login sub-module is a key component of the RMS visualization system, primarily responsible for verifying user identity, maintaining user sessions, and assigning access permissions. By verifying credentials such as usernames and passwords, it ensures that only authorized users can access the system, thereby protecting data security. The login procedure is as follows: first, navigate to the remote support module page; second, enter the corresponding user credentials; finally, click the login button. The specific functional process, module display, and interface design are detailed in Figure 8 and Table 5.

(2) Remote support collaboration sub-module

**Figure 9:**
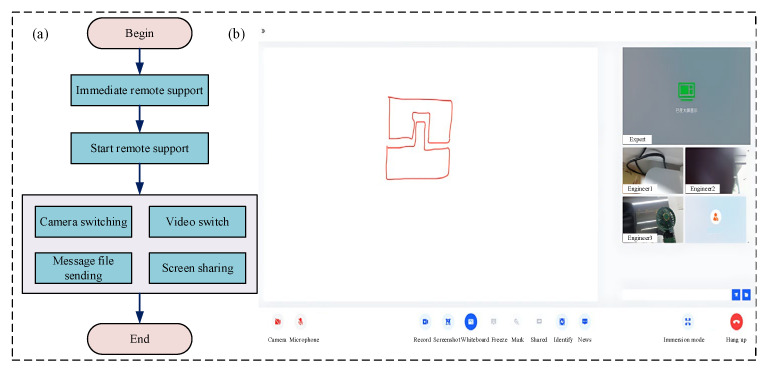
Remote support collaboration sub-module: (**a**) remote support collaboration sub-module function flowchart; (**b**) remote support collaboration sub-module display diagram.

The remote support collaboration sub-module is primarily designed for remote collaboration between maintenance technicians and technical experts. Its feature set includes camera and microphone control, session recording, screen sharing, instant messaging, member invitation, call termination, and file transfer capabilities. It satisfies key performance requirements, including low-latency real-time audio/video streaming and messaging, secure encrypted data transmission, and low resource consumption. The procedure to initiate remote support is as follows: Firstly, select the ‘Immediate Remote Support’ button. Secondly, perform the RMS task utilizing real-time collaboration features such as messaging and file sharing. Finally, terminate the session and exit the collaboration interface. The specific functional process, module display, and interface design are detailed in Figure 9 and Table 6.

(3) Role management sub-module

**Figure 10:**
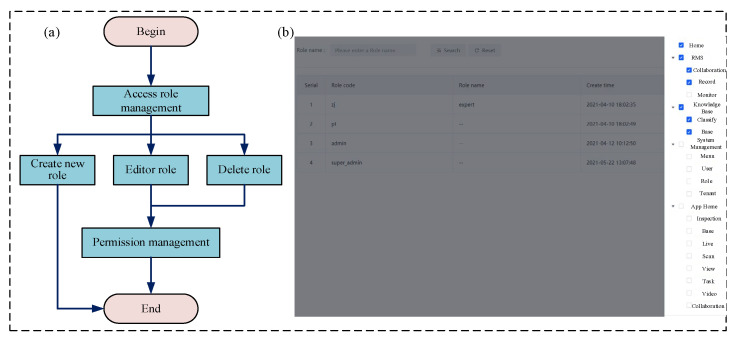
Role management sub-module: (**a**) role management sub-module function flowchart; (**b**) role management sub-module display diagram.

The role management sub-module serves as the core component of the access control system, enabling the centralized definition, maintenance, and assignment of system roles and their associated permissions. This mechanism facilitates granular and dynamic permission control, which effectively mitigates role conflicts and operational inconsistencies during collaborative maintenance tasks. For instance, maintenance technicians may be granted permission to view equipment status and submit repair work orders but are restricted from accessing cost-related data in the spare parts inventory. In contrast, managerial personnel possess extended permissions that include work order assignment and maintenance record auditing. The specific functional process, module display, and interface design are detailed in Figure 10 and Table 7.

### 3.2. RMS Case Study

The vehicular power and energy system is a crucial component of complex equipment. Owing to its compact and highly integrated structure, the new power system presents significant maintenance challenges for personnel. To address these maintenance challenges, this study proposes an IAR-RMS method. This approach implements a remote maintenance support model to enable rapid power system maintenance, enhancing operational efficiency, shortening troubleshooting duration, and reducing errors caused by operator missteps or misinterpretations.

This study employs the disassembly and assembly of a flywheel housing within a power system as a representative maintenance task. A 3D-printed model replicating an actual power system serves as the experimental platform. Participants comprise on-site maintenance technicians using ARs and remote experts operating via PCs. The complete remote support process for flywheel housing maintenance is documented from three perspectives: the maintenance technician first-person view, a third-party observer view, and the remote expert first-person view. The remote maintenance procedure consists of four sequential steps: (1) initiating remote support and establishing expert connection, (2) obtaining remote guidance and technical information, (3) performing maintenance operations and recording key steps, and (4) completing information sharing and exiting support.

(1) Starting RMS and connecting with experts

**Figure 11:**
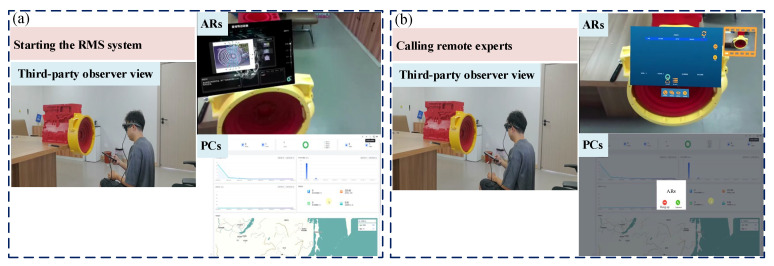
Starting RMS and connecting with experts: (**a**) starting the RMS system; (**b**)calling experts.

Firstly, maintenance technicians disassemble and reassemble the power system in accordance with the steps in the maintenance guide. If they encounter any difficulties while disassembling the power energy flywheel housing, they activate the RMS system. Then, they select ‘start collaboration’ on the RMS system and call a remote expert. Finally, they communicate with the remote maintenance expert via the RMS system to discuss the maintenance details, as shown in Figure 11.

(2) Obtaining remote guidance and technical information

**Figure 12:**
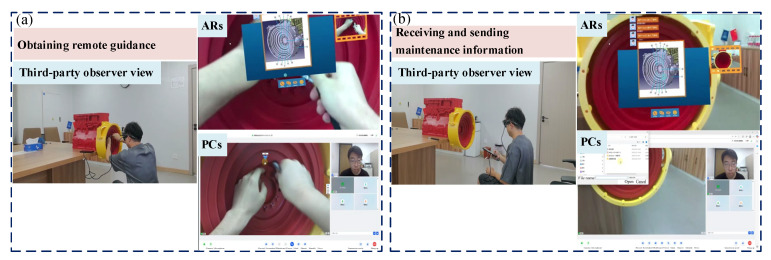
Obtaining remote guidance and technical information: (**a**) obtaining remote guidance; (**b**) receiving and sending maintenance information.

Firstly, the RMS system utilizes real-time audio and video feeds to provide remote experts with a visual assessment of the flywheel housing component failures, awaiting their diagnostic assessment. Secondly, on-site technicians receive step-by-step operational guidance from the remote experts and view the detailed maintenance information provided. Finally, prior to executing the flywheel housing maintenance procedures, maintenance technicians confirm key steps with experts, as shown in Figure 12.

(3) Performing maintenance operations and recording key steps

**Figure 13:**
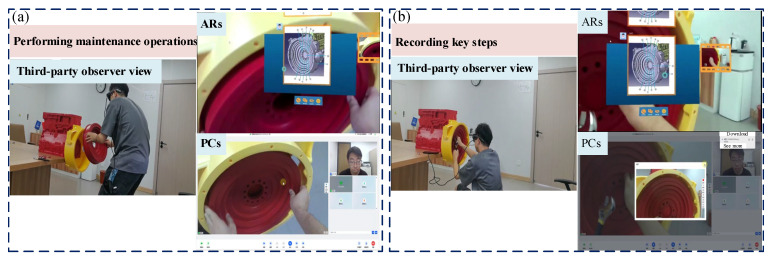
Performing maintenance operations and recording key steps: (**a**) performing maintenance operations; (**b**) recording key steps.

Firstly, maintenance technicians disassemble the flywheel housing according to remote guidance from experts and maintenance technical documentation. This process primarily involves sequentially loosening the M10 × 45 bolts, removing the starter motor, loosening the M10 × 40 bolts, and finally removing the flywheel and its housing. Secondly, the remote experts record the key operational steps of the maintenance process and save them in separate documents for verification. Finally, upon completion of the repair steps, both parties collaboratively inspect the flywheel housing and conduct preliminary functional tests, as shown in Figure 13.

(4) Completing information sharing and exiting support

**Figure 14:**
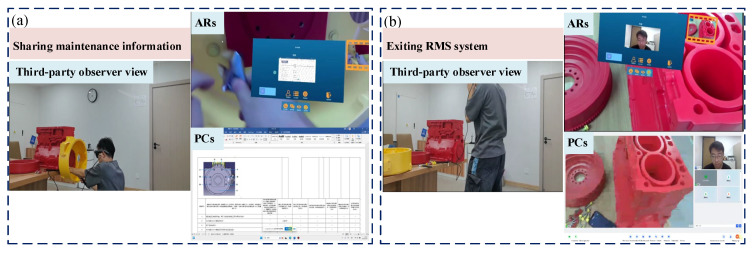
Completing information sharing and exiting support: (**a**) sharing maintenance information; (**b**) exiting RMS system.

Firstly, the remote experts share follow-up maintenance information and communicate with the on-site technicians to provide further guidance. Secondly, the on-site technicians and the remote expert jointly confirm the completion of the flywheel housing dismantling process. The relevant documentation is then archived. Finally, both parties exit the remote support module, power down the audiovisual equipment, and terminate the session, as shown in Figure 14.

To evaluate the effectiveness of the proposed IAR-RMS method, this study conducted experiments involving two maintenance tasks: disassembly of the flywheel housing and the complete disassembly and reassembly of a power and energy system. Five participants with no prior maintenance experience were selected to perform the operations using both the IAR-RMS and the interactive electronic technical manual (IETM) methods. During the experiments, the task completion time, number of procedural errors, and satisfaction level were recorded for each participant. The average values across all five participants were calculated to derive three quantitative metrics: mean maintenance time, error rate, and user satisfaction score. User satisfaction was assessed using a five-point Likert scale.

**Figure 15:**
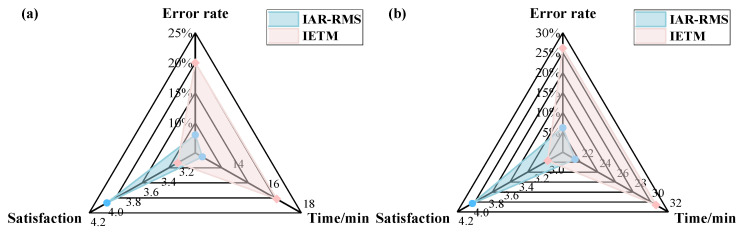
Performance comparison of IAR-RMS and IETM methods on the power system: (**a**) flywheel housing disassembly; (**b**) full disassembly and reassembly.

As shown in Figure 15, the experimental results indicate that in two typical maintenance tasks—namely, disassembly of the flywheel housing and complete disassembly and reassembly of the powertrain energy system—the proposed IAR-RMS method significantly outperformed the conventional IETM method. It achieved efficiency improvements of 25.3% and 30.1%, along with error rate reductions of 12% and 20%, respectively. These outcomes reflect substantial enhancements in both maintenance efficiency and operational accuracy. Moreover, user feedback indicated markedly higher satisfaction with the IAR-RMS method. In summary, the IAR-RMS method demonstrates strong overall performance and considerable application potential in terms of maintenance efficiency, operational accuracy, and user acceptance.

## 4. Conclusions

This paper proposes a remote maintenance support method for complex equipment, based on a layered-MVC-B/S integrated AR framework, to enable multi-person collaborative scenarios. Firstly, guidance information and maintenance processes in remote support operations are analyzed to build a knowledge foundation. Subsequently, resolution strategies are proposed to address role conflicts and operational conflicts that arise during multi-user collaborative maintenance. An RMS model based on the layered-MVC-B/S integrated architecture is established to ensure system reliability and real-time performance during multi-user collaborative tasks. Finally, the effectiveness of the proposed IAR-RMS method is validated using a case study involving the disassembly and assembly of a flywheel housing in a power system.

(1) An RMS process design and analysis methodology is developed; maintenance guidance information is systematically categorized; a comprehensive process framework for the entire task is established; and effective management of process interaction events is implemented.
(2) A collaborative management scheme for RMS is developed to address maintenance conflicts from two dimensions: role conflicts and operational conflicts. Permission allocation and scheduling management strategies are devised to mitigate conflict risks during multi-person synchronous operations.
(3) An RMS model based on a layered-MVC-B/S integrated architecture is established to ensure system reliability and real-time performance in multi-user collaborative maintenance tasks.

Experimental results demonstrate that the proposed IAR-RMS method significantly enhances efficiency and reliability in multi-user remote collaboration tasks while ensuring robust maintenance of complex equipment. However, the proposed IAR-RMS method has limitations. First, the current validation is based on a limited sample size, which may be insufficient to detect significant effects reliably. Second, the method lacks full integration with advanced artificial intelligence (AI) technologies, such as large language models, which restricts its potential for enhancing intelligent capabilities. Future work will include larger-scale experiments with rigorous designs to improve the reliability of the method’s outcomes. Additionally, future efforts will focus on integrating AI and digital twin technologies to enhance the intelligence and adaptability of the proposed IAR-RMS method.

## Figures and Tables

**Table 1:** Typical maintenance guidance information categories.

Category	Sub-Category	Definition	Examples
Physical parameters	Dimensions	3D Geometric Features	Length, width, height
	Material	Material classification and fundamental properties	Plastics, stainless steel
Structural components	Fasteners	Standard mechanical fasteners	Bolts, nuts
	Bearings	Rotational constraint assemblies	Deep groove ball bearings
	Seals	Containment components	Sealing covers
	Electrical components	Electro-power conversion	Batteries, relays
	Modular assemblies	Field-replaceable units	Motors, filters
Maintenance content	Maintenance methods	In situ or depot maintenance	On-site maintenance, off-site maintenance
	Maintenance levels	Maintenance echelon levels	Depot level, intermediate level, organizational level
	Maintenance task types	Technical maintenance categories	Replacement, repair, inspection
	Maintenance actions	Ergonomic postures	Position, posture
Maintenance resources	Maintenance technicians	Maintenance technicians	Maintenance technicians
	Maintenance tools	Maintenance tools	Torque wrenches
	Spare Parts support	Spare Parts support	Modular spares
	Maintenance site	Maintenance site	Repair facilities, workshops
Fault condition	Failure mode	Failure mechanism	Fracture, seal failure
	Failure phenomenon	Failure manifestation	Cracks, oil leakage
	Correlated failure	Cascading failure	Cascading failure from overload
	Diagnostic criteria	Maintenance verification metrics	Performance parameters

**Table 2:** Maintenance content of the RMS.

Category	Sub-Category	Definition
Maintenance methods	On-site maintenance	Repairs carried out at the original location of the equipment
	Off-site maintenance	Repairing equipment at a special facility
Maintenance levels	Organizational level	Basic maintenance performed on-site by front-line personnel
	Intermediate level	Professional maintenance at the regional support level
	Depot level	Extensive repairs and renovations carried out on key facilities
Maintenance task types	Resource preparation	The process of preparing tools and other repair resources
	Troubleshooting	Determine the root cause of the fault through testing
	Replacement	Replacing a defective part with a new part
	Repair	Repair damaged parts to restore equipment functionality
	Debugging	Adjust equipment parameters and calibrate to standard conditions
	Inspection	Check or test equipment performance to verify status
	Preservation	Rust prevention, lubrication and other protective maintenance
Maintenance actions	Bearing	The action of determining or adjusting the position of equipment
	Attitude	Adjusting the orientation or configuration of equipment

**Table 3:** Maintenance role requirements correspondence table.

	Personnel Type	Maintenance Technicians	Technical Support Staff	Management Personnel	Decision Makers	Evaluators	Analysts
Need or Not							
Human–computer interaction		√	√	√			
Model display		√	√	√	√	√	√
Watch the demo		√	√	√	√	√	√
Retrieval process		√	√				
Identify the fault condition		√	√		√		
Remote assistance/support		√	√				
View repair results		√	√			√	√
Work network construction				√	√		
Communication and exchange		√	√	√			
Actual repair work		√					

**Table 4:** Maintenance role permission planning table.

	Personnel Type	Maintenance Technicians	Technical Support Staff	Management Personnel	Decision Makers	Evaluators	Analysts
Competence							
Model construction				√			
Permission management				√			
Interface integration				√			
Maintenance preparation				√			
Task decomposition					√		
Virtual scene construction				√			
Collaborative management				√			
Procedure demonstration		√	√				
Model interaction		√	√				
Maintenance guidance		√	√				
Fault localization		√	√				
Maintenance data exchange		√	√				
Real-time communication		√	√				
Summary and evaluation						√	√

**Table 5:** Login sub-module interface design.

Request URL: /sys/login				
Request type: POST request				
Request input parameters				
Variable name	Data type	Attribute	Storage class	Description
Username	String	Required	Maximum length: 64 bytes	Username
Password	String	Required	Maximum length: 64 bytes	Project name
Response output parameters				
Success	Bool		True or false	Whether successful
Message	String			Success or error message
Code	String		Success 200/Failure 500	Success/failure ID
Result			Returned data object	
Result-userInfo-realname	String		Real name	
Result-userInfo-username	String		Username	

**Table 6:** Remote support collaboration sub-module interface design.

Request URL: /ycxz-api/goolton/task/startTogether				
Request type: POST request				
Request input parameters				
Response output parameters				
Variable name	Data type	Attribute	Storage class	Description
Success	Bool	Required	True or false	Whether successful
Message	String			Success or error message
Code	String		Success 200/Failure 500	Success/failure ID
Data	Object			
Room code	String		Room	Room code
Room Id	String		2202	Room number
Task Id	String		012202	Task number

**Table 7:** Role management sub-module interface design.

Request URL: /ycxz-api/jsgl				
Request type: POST request				
Request input parameters				
Response output parameters				
Variable name	Data type	Attribute	Storage class	Description
Success	Bool	Required	True or false	Whether successful
Message	String			Success or error message
Code	String		Success 200/Failure 500	Success/failure ID
Data	Object			
Role name	String			Character name
Description	String			Remarks
Role code	String			Character code

## Data Availability

Data are contained within the article.
